# Reducing the kyphosis effect of anterior short thoracolumbar/lumbar scoliosis correction with an autograft fulcrum effect

**DOI:** 10.1186/s12891-021-04083-1

**Published:** 2021-02-23

**Authors:** Mazda Farshad, Andrea Frey, Thorsten Jentzsch, Michael Betz, Jonas Widmer, José Miguel Spirig

**Affiliations:** 1 Spine Division, Balgrist University Hospital, University of Zurich, Forchstrasse 340, 8008 Zurich, Switzerland; 2 Laboratory for Orthopaedic Biomechanics, Balgrist University Hospital, Zürich, Switzerland; 3 Institute of Biomechanics, ETH Zurich, Zurich, Switzerland

**Keywords:** Scoliosis, Kyphosis correction, Anterior scoliosis correction, Lumbar scoliosis, Thoracolumbar scoliosis, Kyphosis prevention

## Abstract

**Background:**

Anterior scoliosis correction is a powerful technique with the disadvantage of a kyphotic effect on lumbar and thoracolumbar curves. We aimed to investigate whether a cognizant interposition of a rib graft anteriorly and at the concave side of the scoliotic curve causes significant fulcrum effect to enforce scoliosis correction and to reduce interfusional kyphosis in anterior scoliosis corrections.

**Methods:**

Twenty otherwise comparable patients with lumbar and thoracolumbar adolescent idiopathic scoliosis (AIS) curves undergoing anterior short scoliosis correction with (*n* = 10) or without (*n* = 10, matched for age, gender and degree of deformity) fulcrum effect were retrospectively compared by means of radiographic measurements (sagittal and coronal profile, Cobb angles and intersegmental deformity correction angles) to evaluate the effect of this modified surgical technique.

**Results:**

The overall amount of scoliosis correction was similar with 74 and 60% of initial curves of 57° and 53° in the case and control group respectively with a mean of 3 fused segments (4 screws).

Statistically relevant differences were found for intersegmental coronal cobb angles at the apex of 20° to 3° and 17° to 9° with and without fulcrum, respectively (*p* < 0.05). Creation of kyphosis in the fused segments was reduced with an interfusional kyphotic sagittal cobb angle of 15° pre-operatively vs. 3° post-operatively compared to the control group (13° pre-operatively vs. 18° post-operatively), (*p* < 0.05).

**Conclusions:**

Interfusional hyperkyphosis associated with anterior scoliosis correction for thoracolumbar/lumbar curves can be reduced with cognizant positioning of the bone autograft at the antero-lateral (concave) site in the intervertebral region to create a fulcrum effect.

**Trial registration:**

Registered at *swissethics*: BASEC No.: 2018–00180.

## Background

Since the introduction of the anterior approach to scoliosis correction by Dwyer [1] and its later modification by Zielke [2], the concept has gained popularity following the introduction of rigid rod implants in the early 1990s [3–8]. The anterior approach to scoliosis correction is associated with shorter fusion distance compared to a dorsal technique, thereby sparing spinal motion segments [9, 10]. Additionally, upper thoracic instrumentation can be avoided with the anterior approach to thoracolumbar curves preventing the need to treat compensatory thoracic curves [11]. The disadvantage of this method is a possible reduction of lordosis or a kyphogenic effect caused by a full discectomy and the subsequently applied compression force for bone on bone fusion. Previous studies have demonstrated radiographic and satisfying clinical outcomes of anterior spine fusions over a short-term follow-up (2–5 years) as well as a longer follow-up (12–23 years) [12–14]. However, one main drawback of anterior scoliosis correction remains the occurrence of hyperkyphosis in up to 40% of patients [15].

To our knowledge there are no reports regarding short anterior fusion using cognizant interposition of a rib autograft at the anterior and concave side of the scoliotic curve thereby creating a fulcrum effect to address the problem of kyphosis creation and enhancing coronal scoliosis correction. Therefore, in this study we report the immediate radiographic results, describe the method of choosing fusion levels as well as the specific technique of this surgical procedure.

## Patients and method

The present study was approved by the local ethics committee (*Swissethics*, BASEC No.: 2018–00180) on research involving humans. Every patient involved in this study gave written informed consent before inclusion.

In this cohort study a retrospective review of a very selective patient population with the diagnosis of adolescent idiopathic scoliosis (AIS) with the major curve deformity located in the thoracolumbar or lumbar spine region was performed. All these patients underwent selective short anterior correction fusion with (*n* = 10, cases) or without (*n* = 10, controls) interbody interposition of bone autograft specifically at the anterior and concave side of the scoliotic curve. The controls were selected from 118 AIS patients to match the cases according to age, Risser stage, sex, type and degree of scoliotic deformity (Table 1).

**Table 1:** Patient demographic data (*n* = 20)

Variable	Measurements (median [IQR])		***P***-value^*^
	Cases (***n*** = 10)	Controls (***n*** = 10)	
Age (y)	15 (3)	16 (3)	0.732
Sex (n)			
Female	9	9	1.000
Males	1	1	
Segments (n)	4 (0)	4 (1)	0.185
Lenke (n)			
5	4	5	0.653
6	6	5	
Risser (n)			
0	0	2	0.361
1	2	1	
2	3	1	
3	1	1	
4	4	3	
5	0	2	

*Abbreviations*: *IQR* (interquartile range), *y* (years)

^*^Wilcoxon rank sum test or Chi-square test

Radiological measurements were performed by an independent observer on EOS radiographs (antero-posterior and lateral radiographs) immediately prior to surgery and 6 weeks postoperatively to determine the direct effect of corrective deformity changes according to the recommendation of the SRS [16]. Cobb angle vertebral levels were determined on the preoperative radiographs with the same levels measured on subsequent radiographs to remain consistent for statistical comparison [17]. In addition, intervertebral Cobb angle and the Cobb angle at the apex of fused levels was measured prior to surgery and postoperatively in coronal and sagittal plane. Coronal balance was measured by lateral displacement of a C7 coronal plumbline from the central sacral vertical line (CSVL) [18].

Sagittal alignment measures included the T1 to T12 thoracic kyphosis and lumbar lordosis (L1 to S1). For regional alignment, apical vertebral translation (AVT) was measured as the distance from the geometrical center of the apical vertebra [19] to the central sacral vertical line. Apical vertebral rotation (AVR) or lowest instrumented vertebra (LIV) rotation was graded according to the Nash-Moe method [20].

### Statistics

Due to mainly non-normal distribution, medians (interquartile ranges [IQR]) are provided. Comparison of measurements was done with the Wilcoxon rank sum test (for continuous data) and Chi-squared test (for categorical data) for unpaired groups and the Wilcoxon signed rank test for paired groups. Stata/IC (version 13.1; StataCorp LP, College Station, TX, USA) was used.

For this study no funding source was required.

### Surgical technique

The main surgical technique is described elsewhere in detail [21]. Here we summarize the main steps and the new modification for creation of the fulcrum effect.

To determine fusion length preoperative antero-posterior, lateral as well as supine-side bending radiographic imaging of the whole spine were obtained for all patients. The most caudal opening disc on the concavity of the lumbar curve was not included into the fusion. The number of vertebrae beginning from the apex of scoliosis to the LIV was determined in antero-posterior imaging, whereby the apex can either be a disc or a vertebra. After determining the number of vertebrae, the same number was added proximal to the apex to determine the proximal arm of instrumentation. The average length of fusion was 4 vertebrae (min. 3– max. 5). The distal end of fusion was L2 in five and L3 in another five patients.

All surgical procedures of the case group were conducted by a single surgeon (*blinded for review purposes*) utilizing the same operative technique. Surgery was performed under continued spinal cord monitoring via SEP and MEP. Following double lung intubation all patients were placed in a lateral decubitus position. In all cases a thoraco-phrenico-lumbotomy, including partial resection of a rib, was conducted to expose the thoracolumbar and lumbar intervertebral discs. Subsequently, a 360-degree removal was accomplished through complete anulotomy and discectomy including the removal of the anterior and the posterior longitudinal ligament. The exposed vertebrae were instrumented using a single titanium screw with bicortical purchase.

**Fig. 1:**
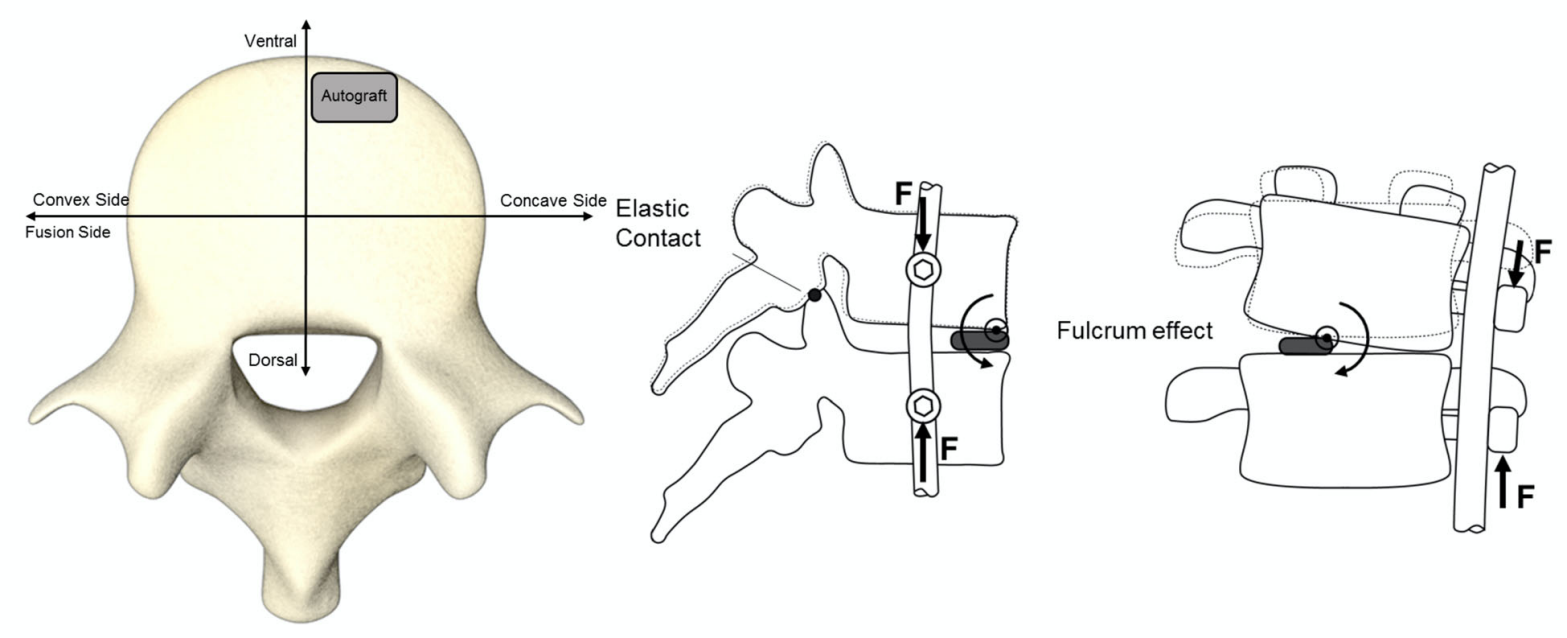
Correct placement of rib autograft (anterolateral at concave side of curve) with segmental fulcrum effect

**Fig. 2:**
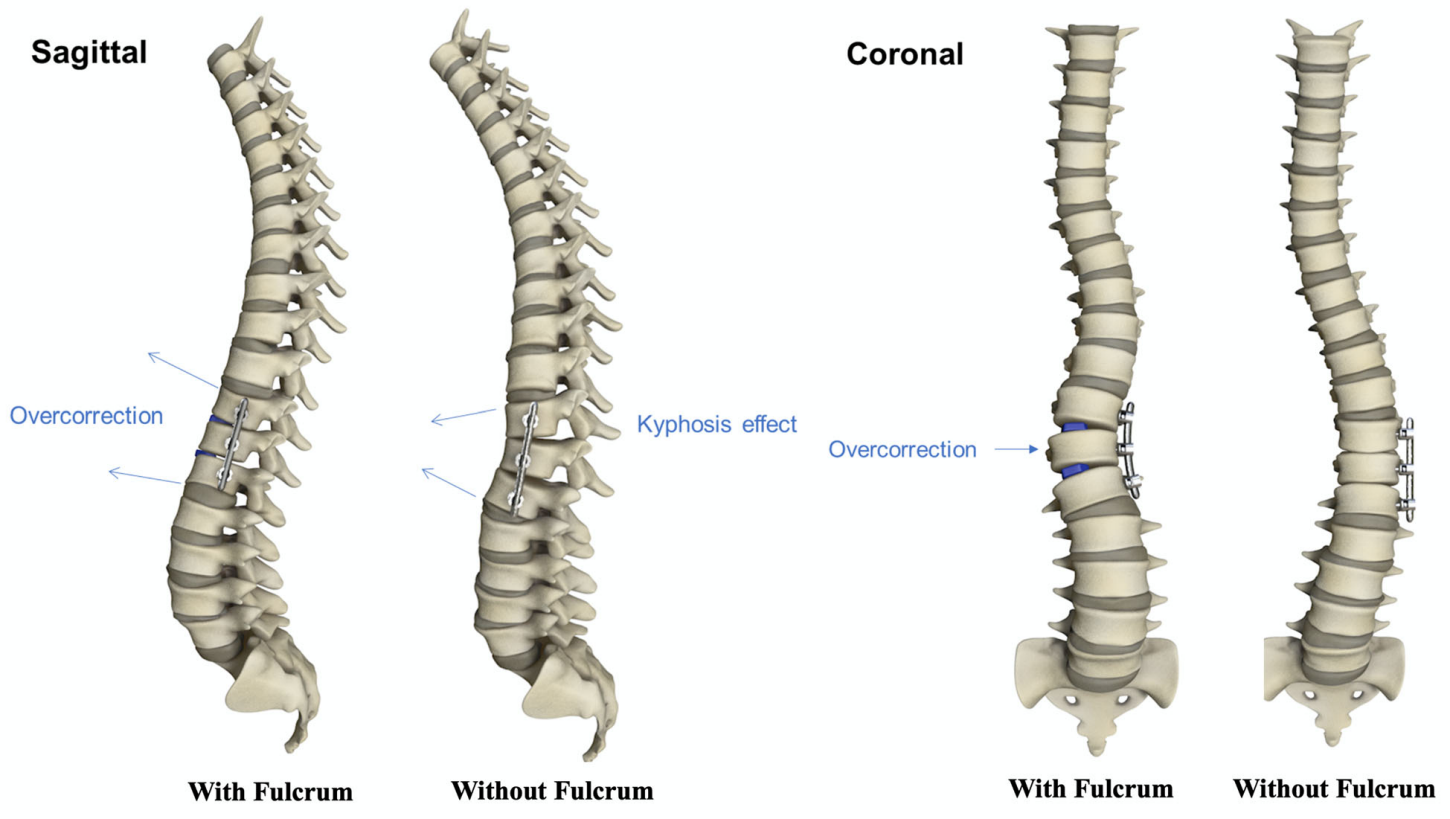
Overcorrection achieved with fulcrum effect in both sagittal and coronal plane. Kyphosis effect seen in sagittal plane without fulcrum

The primarily excised rib was used as structural graft within the intervertebral disc space. Importantly, decortication of the endplates prior to autograft insertion was performed. This increases vascularity and thereby permits speedier osseous healing. The rib autograft (1x1x0,3 cm) was placed between the endplates in the concave curve side to facilitate scoliosis correction and anteriorly to reduce the kyphosis effect created by total discectomy and compression for bone on bone fusion (Fig. 1 and Fig. 2).

A titanium rod was implanted. The rod was fixed to the most proximal screw and subsequently reduced into the distal screws. Subsequently compression forceps were used to achieve final correction aided by the intervertebral rib autograft.

Average surgery time was 237 min with a mean blood loss of 370 ml. Postoperatively all patients were placed in intermediate care for 48 h observation. Ambulation and respiratory therapy were introduced under guidance of physiotherapists on the following day. The thorax drain was removed within 48 – 72 h following surgery. On average patients were dismissed from hospital within 8 days. The first outpatient appointment was performed within 6 weeks of surgery. Six weeks postoperatively, patients were permitted to resume light sportive activities, including cycling and swimming.

No neurological complications, pseudarthrosis or implant-related complications were noted in both groups.

## Results

### Main curve correction

The median pre-operative Cobb angle of the main curve was 57° in the case (IQR 13) and 53° in the control (IQR 12) group. With a mean of 4 fused segments, the scoliotic curves improved by 74% to 12° (IQR 17) immediately post-operatively and remained mostly unchanged with 15° (IQR 16) at 6 weeks in the case group. In the control group fusion was performed over 3.6 segments with a postsurgical median of 20° (IQR 11) and 21° (IQR 10) at 6 weeks post-operatively. This corresponds to a 60% improvement of the scoliosis.

### Apical intervertebral Cobb angle correction

There was a significant difference observed in the intervertebral Cobb angle at the apex of the scoliosis between the case and control group. With a pre-operative median of 20° (IQR 4) the angle improved by 88% to 3° (IQR 2) immediately after surgery. In contrast, the intervertebral Cobb angle in the control group measured 17° (IQR 6) at time of surgery, improving with 53% to 9° (IQR 2) after surgery.

### Apical vertebral translation and coronal balance

Apical vertebral translation and coronal balance were not significantly different between the two groups at each time point.

### Thoracic kyphosis

Thoracic kyphosis changed from a median of 37° (IQR 16) to 36° (IQR 11) 6 weeks postoperatively in the case and from a median of 37° (IQR 11) prior to surgery, increasing to 45° (IQR 15) post-operatively in the control group, respectively.

### Lumbar lordosis

The pre-operative median lumbar lordosis did not decrease significantly from 53° (IQR 20) to 52° (IQR 12) at 6 weeks postoperatively in the case group versus 57° (IQR 7) to 64° (IQR 12) in the control group.

### Apical vertebral rotation

The apical vertebral rotation was neither significant in the case nor in the control group (IQR 1) (Table 2).

**Table 2:** Comparison of standard radiological measurements between cases and controls (*n* = 20)

Variable	Measurements (median [IQR])		
	Cases (***n*** = 10)	Controls (***n*** = 10)	***P***-value^*^
Overall Cobb angle (°)			
Pre-op	57 (13)	53 (12)	0.732
Post-op	11 (17)	20 (11)	0.403
6 wks	15 (16)	21 (10)	0.427
Δ (pre-op vs 6 wks)	−40 (11)	−36 (12)	0.448
AVT (mm)			
Pre-op	45 (15)	41 (19)	0.140
Post-op	11 (7)	15 (11)	0.383
6 wks	14 (12)	18 (4)	0.307
Δ (pre-op vs 6 wks)	−32 (15)	−21 (16)	0.028^†^
Coronal balance (mm)			
Pre-op	24 (4)	22 (10)	0.197
6 wks	23 (4)	19 (7)	0.139
Δ (pre-op vs 6 wks)	−1.5 (6)	−2.5 (10)	1.000
Apical intervertebral Cobb angle (°)			
Pre-op	20 (6)	17 (6)	0.363
Post-op	3 (2)	9 (2)	< 0.001^†^
6 wks	2 (1)	9 (2)	< 0.001^†^
Δ (pre-op vs 6 wks)	−18 (8)	−10 (8)	0.002^†^
Thoracic kyphosis (°)			
Pre-op	37 (16)	37 (11)	0.910
6 wks	36 (11)	45 (15)	0.064
Δ (pre-op vs 6 wks)	−1 (6)	9 (7)	0.010^†^
Lumbar lordosis (°)			
Pre-op	53 (20)	57 (7)	0.544
6 wks	52 (12)	64 (12)	0.023^†^
Δ (pre-op vs 6 wks)	−4 (7)	3 (6)	0.017^†^
Apical vertebral rotation (Nash and Moe Grade)			
Pre-op	2 (1)	2 (1)	1.00
6 wks	2 (1)	2 (1)	0.374
Δ (pre-op vs 6 wks)	−1 (1)	−1 (1)	0.593

*Abbreviations*: *IQR* (interquartile range), *n* (number), *°* (degrees), *AVT* (apical vertebral translation) *mm* (millimeters), *op* (operatively), *wks* (weeks), *vs* (versus), *Δ* (delta [difference])

^*^Wilcoxon rank sum test or Chi-square test

^†^Statistically significant difference

### Coronal Cobb angle at the level of instrumentation

The coronal Cobb angle at the level of instrumentation improved from a median of 45° (IQR 19) to 5° (IQR 12) 6 weeks postoperatively in the case group compared to 36° (IQR 9) to 14° (IQR 10) in the control group.

### Sagittal Cobb angle at the level of instrumentation

In the sagittal profile the case group showed a statistically relevant reduction of kyphotic angle within the instrumented levels with a median of 14° (IQR 6) preoperatively to 3° (IQR 8) 6 weeks postoperatively whereas in the control group an increasement of kyphosis was observed from 13° to 18°.

### Thoracic kyphosis above instrumentation

The thoracic kyphosis above instrumentation changed non significantly from a median of 14° (IQR 5) to 12° (IQR 6) 6 weeks postoperatively in the case group and from 22° (IQR 8) to 19° (IQR 19) post-operatively in the control group.

### Lumbar lordosis below instrumentation

The pre-operative median lumbar lordosis below the level of instrumentation decreased from 47° (IQR 23) to 39° (IQR 19) at 6 weeks postoperatively in the case group whereas a substantial subfusional, compensatory increasement in lordosis from 42° (IQR 14) to 45° (IQR 12) was measured in the control group (Table 3).

**Table 3:** Comparison of interfusional and sub- and epifusional radiological measurements between cases and controls (*n* = 20)

Variable	Measurements (median [IQR])		
	Cases (***n*** = 10)	Controls (***n*** = 10)	***P***-value^*^
Coronal Cobb angle at instrumented level (°)			
Pre-op	45 (19)	36 (9)	0.129
Post-op 6 wks	5 (12)	14 (10)	0.058
Δ (pre-op vs 6 wks)	−41 (5)	−24 (15)	0.002^†^
Sagittal Cobb angle at instrumented level (°)			
Pre-op	14 (6)	13 (11)	0.363
Post-op 6 wks	3 (8)	18 (18)	0.006^†^
Δ (pre-op vs 6 wks)	−12 (11)	5 (4)	0.001^†^
Epifusional sagittal Cobb angle at non-instrumented level (°)			
Pre-op	14 (5)	22 (8)	0.023^†^
Post-op 6 wks	12 (6)	19 (19)	0.211
Δ (pre-op vs 6 wks)	−2 (2)	−6 (6)	0.087
Subfusional sagittal Cobb angle at non-instrumented level (°)			
Pre-op	47 (23)	42 (14)	0.762
Post-op 6 wks	39 (19)	45 (12)	0.112
Δ (pre-op vs 6 wks)	−4 (7)	6 (5)	0.006^†^

*Abbreviations*: *IQR* (interquartile range), *n* (number), *°* (degrees), *op* (operatively), *wks* (weeks), *vs* (versus), *Δ* (delta [difference])

^*^Wilcoxon rank sum test or Chi-square test

^†^Statistically significant difference

## Discussion

The main disadvantage of a short anterior lumbar scoliosis correction is its potential kyphogenic effect. We investigated whether interbody cognizant interposition of bone autograft at the anterior and concave side of the scoliotic lumbar and thoracolumbar curve would reduce the undesired kyphogenic effect. We found that the described technique seems powerful in this regard, despite technically simple.

An anterior approach to scoliosis correction was first described by Dwyer in 1969 [1, 22]. His technique included resection of the discs and applying compressive forces with a cable and screw system. The major drawback of this technique was a limited stability since the flexible cable still allowed some amount of motion. Therefore, implant failure and subsequent pseudoarthrosis were frequent complications with this technique [23–25].

**Fig. 3:**
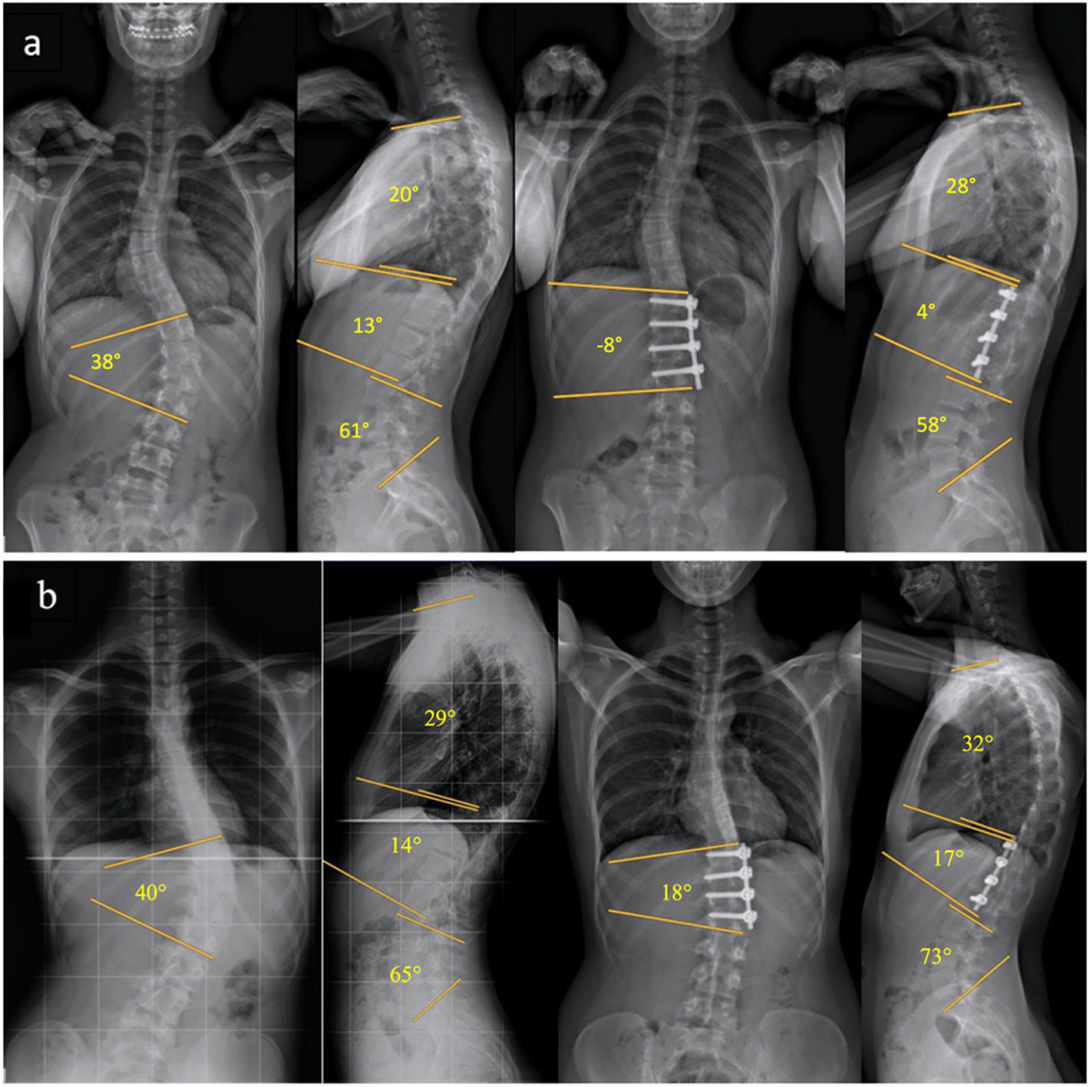
**a** Standing PA and lateral radiographs with pre- and postoperative Cobb angles in a patient operated with autograft fulcrum effect. **b** Standing PA and lateral radiographs with pre- and postoperative Cobb angles in a control patient operated without autograft fulcrum effect

In 1976 the surgical technique was modified by Zielke substituting the wires with threaded rods (a Harrington compression rod) to connect the implanted screws [2]. This provided more stability against rotational forces and allowed to apply some derotational maneuver during surgery. Later on, Hall and Bernstein introduced a surgical method of shorter fusion using Zielke implants in adolescents with flexible idiopathic thoracolumbar scoliosis [3, 5]. They emphasized the importance of overcorrection in short anterior correction to achieve acceptable results. Since the early 1990s, following the introduction of rigid screw-rod implants, there have been numerous reports on the anterior correction of thoracolumbar/ lumbar scoliosis [4–8]. However, one main drawback of anterior scoliosis correction remains the occurrence of hyperkyphosis in up to 40% of patients by applying anterior compression forces [15]. Whilst other studies have described the use of cages for anterior correction of thoracolumbar/lumbar curves to maintain lordosis [10, 26] there is no study evaluating the strategic important positioning of a structural interbody bone autograft at the anterior and concave side of the scoliotic curve to introduce a fulcrum and counteract the kyphogenic effect of anterior scoliosis correction. (Fig. 3a and Fig. 3b).

However, the use of rib autograft has been described earlier [4, 5, 9] and is a known factor in contributing to rapid fusion.

We could clearly show that while those patients, in whom the autograft fulcrum effect was used, demonstrated a correction of preoperative thoracolumbar/lumbar kyphosis at the instrumented levels (14° to 3°), the control group showed a clear increase in kyphosis (13° to 18°). Kelly et al. [4] who used rib autograft without specifically describing the positioning of the autograft showed a significant increase in kyphosis from 2° to 11° in the instrumented levels. However, similar to our study, they instrumented 3 to 4 segments and they had no significant change in global thoracic kyphosis and lumbar lordosis from pre- to postoperative. Measurements for lumbar lordosis and thoracic kyphosis were performed at different levels than in our study wherefore comparison of absolute numbers are not reasonable. Bernstein et al. [5] who used rib autograft without specific positioning for fusion of 2 to 3 segments reported similarly a significant increase in kyphosis from 4° to 10° within instrumented levels. Measurements of global thoracic kyphosis and lumbar lordosis were not reported. Min et al. [9] fused on average 3 segments using also an unspecific rib autograft technique and reported no significant change in global thoracic kyphosis and lumbar lordosis. Unfortunately, no measurements of cobb angle in the instrumented segments were performed. Overall, it is reasonable that the amount of creation or reduction of kyphosis is somehow depending on the exact positioning of the rib autograft; While a dorsal positioning plausibly does not reduce kyphosis, an anterior intervertebral position seems to reduce the kyphosing effect of anterior scoliosis correction. This was also observed in our case group using the rib autograft fulcrum effect.

In addition, the lateral concave side positioning allows an even stronger coronal correction than the bone-on-bone fusion technique allowing shorter fusion segments for over-correction of the apex, potentially. However, this correction difference (74% versus 60%) was not significant in our study. The correction of the case group in our study was comparable with results of other studies using an unspecific rib autograft technique (87% by Bernstein et al. [5], 64% by Kelly et al. [4], 67% by Min et al. [9]).

The results of this study, however, need to be interpreted with regard to their limitations.

First, this was a retrospective analysis of a limited number of patients. However, in anticipation of this limitation and in order to increase the validity of the study, case-control matching was carried out. Due to homogeneity of the comparative groups the strength of the study was thereby increased.

Second, a 6 week follow-up is not enough for a surgical outcome study. However, we specifically report the immediate results of a modified surgical technique and do not claim to report any associated clinical outcome measures or long term follow up. We believe that the promising early results of this surgical technique which eliminates a major limitation (creation of kyphosis) of an otherwise advantageous surgical procedure for correction of scoliosis (anterior short correction) might be of interest to the spine surgery community. The modification described here is based on the established technique of bone-autograft interposition, but with a cognizant strategic positioning.

Third, the radiological assessment of the scoliosis and its correction was made on ap and lateral radiographs and not 3D-enabled imaging. While we believe, that the fulcrum effect might be even more valuable if the evaluations were made in a 3D-modality imaging, this hypothesis remains untested.

## Conclusion

Based on this study we conclude that anterior short fusion using antero-, concave-lateral positioning of a rib autograft as a fulcrum is a valuable technical addition for correction of thoracolumbar/lumbar idiopathic scoliosis. In addition to reduction of the otherwise occurring kyphosis effect with anterior scoliosis correction, a better short (over-) correction of the apex seems possible. Whilst the initial results show promising results, follow-up studies will be necessary to validate long term results and demonstrate preservation of the achieved correction.

## Data Availability

The datasets used and/or analyzed during the current study are available from the corresponding author on reasonable request.
